# Metronomic chemotherapy: changing the paradigm that more is better

**DOI:** 10.3747/co.v16i2.420

**Published:** 2009-03

**Authors:** O.G. Scharovsky, L.E. Mainetti, V.R. Rozados

**Affiliations:** * Instituto de Genética Experimental, Facultad de Ciencias Médicas, Universidad Nacional de Rosario, Rosario, Argentina

**Keywords:** Metronomic chemotherapy, angiogenesis, optimal biologic dose, obd, circulating endothelial cells, cecs, circulating endothelial progenitors, ceps

## Abstract

The introduction of the “maximum tolerated dose” in usual treatment protocols (and its concomitant overt toxicity) made necessary the imposition of rest periods between cycles of therapy—a practice that not only involves re-growth of tumour cells, but also growth of selected clones resistant to the therapy. To avoid the problems caused by traditional chemotherapeutic regimens, a new modality of drug administration called “metronomic chemotherapy” has been proposed. This name makes reference to the chronic, equally spaced administration of (generally) low doses of various chemotherapeutic drugs without extended rest periods. The novelty of this treatment modality lies not only in its antitumour efficacy with very low toxicity, but also in a cell target switch, now aiming at tumour endothelial cells. The knowledge acquired in the experimental field of metronomic chemotherapy, plus the increasing experience that is being obtained in the clinical setting, will help to lead a change in the design of therapeutic protocols against cancer.

## 1. INTRODUCTION

Chemotherapy regimens reflect a controversy that is by now historical: between efficacy in tumour killing and lack of toxicity, which way should the scale be tipped? On one side is the ability of chemotherapeutic drugs to disrupt the dna of tumour cells, rendering them unable to replicate and finally killing them, with a befitting corollary: “the higher the dose, the better.” On the other side is the toxicity expressed at several organ sites, which not only diminishes quality of life for the patient, but also conspires against a good resolution of the cancer treatment, adding more illness to the already existing one. The introduction of the maximum tolerated dose (mtd) in usual treatment protocols made necessary the imposition of rest periods between cycles of therapy—a practice that not only involves re-growth of tumour cells, but also growth of selected clones resistant to the therapy. Hence, the therapeutic success obtained during the first cycles of treatment reverts in the direction of growth of more malignant metastatic tumours with no therapeutic response.

## 2. DISCUSSION

### 2.1 A New Therapeutic Philosophy

A turning point in cancer chemotherapy can be placed in the year 2000, when Fidler and Ellis said, “Cancer is a chronic disease and should be treated like other chronic diseases” 1. Simultaneously, and to avoid the problems caused by the traditional chemotherapeutic treatments, several groups, including ours, began to study a new modality of drug administration that Douglas Hanahan called “metronomic therapy” 2. That name makes reference mainly to the schedule, which consists of chronic, equally spaced, and (generally) low doses of various chemotherapeutic drugs without extended rest periods. The first researchers involved in such a proposal, which meant a change in the rationale with which chemotherapy is undertaken, were Judah Folkman and Robert Kerbel and their respective colleagues 3,4. These groups demonstrated the antitumour efficacy of some of the most widely used chemotherapeutic drugs administered chronically in low doses as anti-angiogenic agents, therefore implying a different cell target. This new concept includes the possibility of treating tumours that no longer respond to traditional chemotherapy. The novelty consisted of a cell target switch (aiming at the tumour endothelial cells), together with a change in the schedule and dose of drug administration.

### 2.2 Metronomic Chemotherapy in the Experimental Setting

As with any other experimental therapy, metronomic chemotherapy (mct) built its foundations with plenty of experimental work, beginning with the pioneering work in the Folkman and Kerbel laboratories. Browder and colleagues demonstrated that standard chemotherapeutic drugs such as cyclophosphamide can also be used as anti-angiogenic agents. The administration of cyclophosphamide in doses lower than the mtd, at shorter intervals and without extended rest periods, showed results better than those obtained with the mtd schedule in the treatment of two cyclophosphamide- resistant tumours, Lewis lung carcinoma and the murine mammary carcinoma cell line EMT-6 3. They also eradicated the drug-sensitive Lewis lung carcinoma and the L1210 leukemia using the same therapy. This schedule of cyclophosphamide administered in combination with a specific anti-angiogenic agent (TNP-470) eliminated most drug-resistant Lewis lung carcinomas. Nevertheless, despite being lower than the mtd, the dose of cyclophosphamide was still high, and the experimental animals received palliative care to ameliorate gastrointestinal dysfunction and weight loss.

At almost the same time, Klement *et al.* published important work testing the effect of low-dose continuous chemotherapy as a possible anti-angiogenic strategy. Before undertaking *in vivo* experiments, they established, *in vitro*, a dose of vinblastine that showed antiproliferative effect on human umbilical vein endothelial cells, but not on human neuroblastoma cell lines (SK-NM-C and SK-N-AS). In the *in vivo* model, animals bearing xenografts of both neuroblastoma cell lines were treated with low doses of vinblastine; or a monoclonal neutralizing antibody (DC101) that blocks the function of vascular endothelial growth factor receptor 2 (vegfr2) and, hence, vascular endothelial growth factor (vegf) itself; or both agents together. The animals treated with either drug alone showed inhibition of tumour growth, an effect that did not last. On the other hand, the combination treatment induced an initial response similar to that in the other treatment groups, and that response was followed by long-term tumour regression. The usual signs of drug toxicity in mice (for example, weight loss, ruffled fur, anorexia, cachexia, skin tenting, skin ulceration, or toxic death) were not seen at the doses used in the experiment 4. Vacca and colleagues also used vinblastine during *in vitro* testing of the effect of non-cytotoxic doses on endothelial cell functions involved in angiogenesis and then, *in vivo*, with the chick embryo chorioallantoic membrane model. They showed dose-dependent anti-angiogenic activity 5.

These seminal experiments suggested the possibility that various kinds of tumours may be amenable to chronic treatment, prompting our group, and others, to study the efficacy of metronomic chemotherapy in other tumour models. We focused our interest both on the antitumour and anti-metastatic effect achieved, and on the toxicity of the treatment. We were able to demonstrate that metronomic administration of cyclophosphamide at low doses on a 3-times-weekly schedule eradicated established rat lymphomas and sarcomas (10 mg/kg and 5 mg/kg body weight respectively). Neither metastatic growth nor recurrence at primary sites occurred for 100% of the lymphomas and 83% of the sarcomas, and no weight loss was observed. At the same time, the regimen was devoid of hematologic, cardiac, hepatic, and renal toxicity 6.

The experimental work developed in several laboratories showed antitumour efficacy and a lack of the manifest toxicity of mct in various *in vivo* tumour models in which animals were treated metronomically with diverse agents. Established orthotopic multidrug-resistant human breast cancer xenografts in scid (severe combined immunodeficiency) mice were treated with paclitaxel, vinblastine, cisplatin, or doxorubicin alone or in combination with DC101 7. Also, DC101 was combined with oral cyclophosphamide in a similar model 8 and with doxorubicin to treat a human soft-tissue sarcoma xenograft in scid mice 9. Metronomic cyclophosphamide was also used in a mouse model of pancreatic cancer in combination with another vegfr2 inhibitor (SU5416) or two different inhibitors of matrix metalloproteinases 10. In addition, a combination regimen with metronomic cyclophosphamide plus uft (a 5-fluorouracil pro-drug) showed therapeutic efficacy in a model of advanced metastatic breast cancer 11. Interestingly, the administration of metronomic cyclophosphamide plus low-dose pegylated liposomal doxorubicin achieved a high antitumour effect in an experimental mouse model of metastatic pulmonary melanoma 12. Also, and very importantly, metronomic administration of low-dose cyclophosphamide was demonstrated to show not only good therapeutic results, but also low-grade or absent toxicity in tissues highly sensitive to the toxic effects of mtd regimens 6,13. Several common chemotherapeutic drugs such as vinblastine, carboplatin, topotecan, and cisplatin were tested either alone or in combination with anti-angiogenic agents for the treatment of various human tumours such as glioblastoma, Wilms tumour, breast cancer, and testicular germ cell tumour 14–17. Interesting work showed the efficacy of using both metronomic therapy and standard-of-care mtd in a so-called chemo-switch regimen, suggesting a potential clinical strategy in which standard mtd is followed by a novel maintenance regimen 18.

More recently, a new therapeutic approach was proposed, now targeting the multicellular biologic entity of the tumour microenvironment. Blansfield and colleagues 19 administered metronomic cyclophosphamide plus lenalidomide, an immunomodulatory drug, and sunitinib, a tyrosine kinase inhibitor. This three-drug combination delayed almost completely the progression of tumour growth in xenograft models of ocular melanoma, colon cancer, pancreatic cancer, and cutaneous melanoma.

### 2.3 How Does Metronomic Chemotherapy Work?

Angiogenesis is a normal process that has essential roles in development, reproduction, and tissue repair. On the other hand, pathologic angiogenesis is closely involved in diabetic retinopathy, chronic inflammation, and tumour formation. Angiogenesis plays a critical role in the growth and metastatic spread of tumours 20. The angiogenic switch occurs when levels of angiogenesis stimulators such as vegf and basic fibroblast growth factor (bfgf) exceed those of angiogenesis inhibitors such as thrombospondin-1 (tsp-1) 21,22.

In 1971, Judah Folkman first articulated the concept of what he called “anti-angiogenic” drugs. Based on observations that expansion of a tumour mass was limited in the absence of angiogenesis, he proposed that treatment with drugs that prevent the formation of tumour blood vessels might be able to constrain cancer for prolonged periods 23. Since then, several direct or indirect angiogenesis inhibitors such as bevacizumab, sorafenib, and sunitinib 24–27 have been developed, stimulating the proposal of anti-angiogenic therapeutic schedules. It is worth mentioning that inhibition of angiogenesis had already been demonstrated in animal models by several chemotherapeutic drugs such as mitoxantrone and bisantrene, and cyclophosphamide, paclitaxel, and methotrexate 28–31.

The angiogenic polypeptide vegf is detected in many malignant tumours 32. Several *in vitro* and *in vivo* studies demonstrated that vegf can be considered a marker of the angiogenesis process 33–36. Interestingly, a decrease in serum levels of vegf was observed in patients with advanced breast cancer treated with metronomic low-dose cyclophosphamide 37. Similarly, a metronomic chemotherapy regimen of weekly platinum and daily oral etoposide in patients with high-risk non-small-cell lung cancer showed a decrease in vegf levels during treatment 38.

One of the drawbacks for the extended clinical use of mct is the fact that the most effective dose and schedule have yet to be defined. Therefore, with the aim of filling that need, the roles of various surrogate markers of angiogenesis and vasculogenesis were studied to understand whether they can be used to define the anti-vascular activity of certain drugs or drug combinations, and to predict response or survival in cancer patients receiving anti-angiogenic treatment 39,40. Pro-angiogenic and anti-angiogenic serum proteins such as, respectively, vegf and tsp-1 might act as surrogate markers for monitoring treatment activity 41. In children with recurrent refractory solid tumours treated with celecoxib and metronomic vinblastine or cyclophosphamide, Stempak and colleagues observed substantial inter-patient variability, but no significant relationship between stable disease or disease progression and the serum concentrations of these markers during the course of therapy. These findings are not entirely unexpected, because a vast number of inducers and inhibitors act in concert to tightly regulate angiogenesis 42. Also, in a phase ii study of mct with etoposide and cyclophosphamide in combination with daily thalidomide and celecoxib for recurrent malignant gliomas in adults, the serum and urine levels of vegf, bfgf, endostatin, and tsp-1 were evaluated. Nonsignificant differences between responders and non-responders were found 43. Colleoni *et al.*, who studied vegf serum concentrations in patients with advanced breast cancer receiving metronomic low-dose oral cyclophosphamide and methotrexate plus or minus thalidomide, obtained opposing results. Compared with baseline levels, mean levels of vegf 2 months after treatment were significantly lower 37. Moreover, serum level of vegf was studied as a prognostic factor in patients with recurrent or metastatic squamous cell carcinoma of the head and neck treated with metronomic paclitaxel. Preliminary results suggested an association between vegf and both response and disease stabilization 44.

A number of recent experimental observations suggest that the growth of some types of cancer may depend on vasculogenesis (that is, progenitor cell–dependent generation of new blood vessels) and not just angiogenesis (that is, mature endothelial cell–dependent generation of new blood vessels). Circulating endothelial cells (cecs) are seldom found in the blood of healthy individuals (an exception is the increase by a factor of 1.5–2 seen in women during the active menstrual phase associated with uterine vascular remodelling). However, cecs are detected in patients with neoplastic, inflammatory, and vascular conditions 39. Mature endothelial cells are thought to originate by sloughing from the vessel wall. On the other hand, the circulating endothelial progenitor cells (ceps) that are considered to be an alternative source for some of the endothelial cells of newly formed blood vessels represent a subset of immature vegfr2- positive cecs also found in peripheral blood 45,46. These cells enter the peripheral blood circulation and subsequently incorporate into distal sites of ongoing sprouting angiogenesis, where they can differentiate into mature endothelial cells 47–49.

The increase of cecs and their cep subsets was demonstrated in some preclinical cancer models 50,51 and also in cancer patients 39. The initial assumption was that, unlike tumour cells, cecs would be chromosomally and genetically normal and, therefore, genetically stable. That assumption was the rationale for the proposal of anti-angiogenic therapy, because it was considered that endothelial cells would probably fail to develop drug resistance 52. However, in some melanoma and liposarcoma xenograft models, tumour-associated endothelial cells were unexpectedly found to have heterogeneous nuclei. Also, a study by Streubel *et al*. in patients with B-cell non-Hodgkin lymphoma with specific genetic aberrations showed that endothelial cells of the cancer microvasculature acquired the same specific chromosomal translocations 53. It is therefore important to characterize cecs and their progenitors with several markers according to the tumour types being tested, so as to use them as clinical markers of illness evolution.

Several *in vitro* and *in vivo* studies showed that activated cecs of newly formed blood vessels are highly and selectively sensitive to very low doses of various chemotherapeutic drugs (methotrexate, paclitaxel, vinblastine, 4-hydroperoxycyclophosphamide, taxanes, doxorubicin, etoposide, and 5-fluoruracil) used alone or in combination with an anti-angiogenic drug 7,46,54,55.

The level of ceps was reported to be usable as a surrogate marker for angiogenesis and, thus, as a biomarker for monitoring targeted anti-angiogenic drug activity 56. Accordingly, the dose that causes the maximum decline in viable ceps can be considered to be the optimal biologic dose 57. High-dose celecoxib and metronomic low-dose cyclophosphamide led to a decline in cecs and their precursors in patients with relapsed or refractory aggressive non-Hodgkin lymphoma, suggesting that this combination may be working by inhibiting angiogenesis, a result that should be validated in a larger patient sample 58. Recently, Mancuso and colleagues studied the correlation between the kinetics of cecs and clinical outcome in patients with advanced breast cancer receiving mct. They found that cecs were a particularly good predictor of disease-free and overall survival after a prolonged follow-up of more than 2 years 59. Nevertheless, patients with refractory solid malignancies treated with a fixed daily dose of 50 mg oral cyclophosphamide or a daily dose of 50 mg etoposide and 400 mg celecoxib given twice daily showed no significant change in levels of cecs and ceps. In accord with that result, no significant clinical activity was demonstrated. Therefore, it is possible that changes in circulating cecs and ceps could be observed in the setting of more effective anti-angiogenic therapies 54. These contradictory results underline the need to determine the best markers of response for each type of tumour.

Hamano *et al.* demonstrated that low-dose cyclophosphamide inhibits tumour growth and induces selective apoptosis of endothelial cells within the tumour vascular bed by up-regulating the endogenous angiogenesis inhibitor tsp-1. As compared with wild-type tumours, tumours treated with low-dose cyclophosphamide showed similar expression of both matrix metalloproteinases and basement membrane– derived angiogenesis inhibitors; tsp-1 expression, on the other hand, was significantly increased 60. Bocci *et al.* reported that protracted *in vitro* exposure of endothelial cells to low concentrations of several anticancer agents, including microtubule inhibitors and an alkylating agent, caused marked induction of tsp-1 gene and protein expression. Conversely, an increase was detected in circulating tsp-1 in plasma of scid mice bearing PC-3 human prostate cancer treated with metronomic low-dose chemotherapy 61. The antitumour and anti-angiogenic effects achieved with the administration of low-dose metronomic cyclophosphamide in wild type animals was also demonstrated to be lost in tsp-1–null mice 61. It was shown that tsp-1 can induce apoptosis of microvascular endothelial cells expressing CD36 62. *In vitro* and *in vivo* experiments showed that mct induces the expression of tsp-1 and that this up-regulation has a negative effect on endothelial cell survival 60,61. Taken together, these results implicate tsp-1 as a secondary mediator of the anti-angiogenic effect of at least some mct regimens.

Another mechanism responsible for the antitumour effect of mct with certain chemotherapeutic drugs could be the stimulation of the immune response, because metronomic administration of oral cyclophosphamide in advanced cancer patients induces a profound and selective reduction in circulating regulatory T cells (Tregs). This effect is associated with suppression of Treg inhibitory functions on conventional T and natural killer cells, leading to restoration of peripheral T-cell proliferation and innate killing activities 63. Furthermore, in an experimental setting, our own results support the notion of the involvement of the immune system in the antitumour effect of mct with cyclophosphamide. Euthymic rats and nude mice bearing a rat B-cell lymphoma were treated metronomically with cyclophosphamide. After a short period of tumour growth, all treated rats showed sustained tumour regression, an effect that none of the non-treated control rats showed. Interestingly, neither treated nor control nude mice showed tumour regression, a result that could be ascribed to the absence of the adaptive immune response 64. As a consequence, the aforementioned data support the notion that a metronomic regimen with cyclophosphamide not only affects tumour angiogenesis, but also exerts its therapeutic effect mediated by the immune system.

**FIGURE 1:**
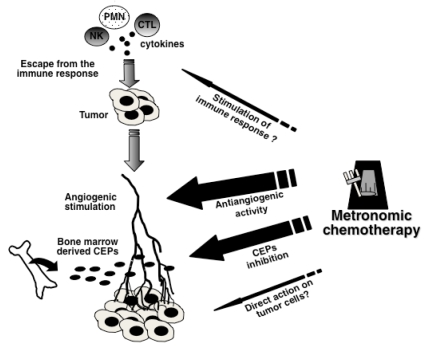
Mechanisms of action operating in metronomic chemotherapy. pmn = polymorphonuclear leukocyte; ctl = cytotoxic T lymphocyte; nk = natural killer cell; ceps = circulating endothelial progenitor cells.

Figure 1 summarizes the mechanisms of action operating in metronomic chemotherapy. Briefly, the two fundamental processes induced by the tumour that enable its growth and progression are angiogenesis (involving the growth of new blood vessels from pre-existing vessels or from ceps) and escape from the immune response. The antitumour and anti-metastatic effects of metronomic chemotherapy would be achieved through several mechanisms, including inhibition of ceps, anti-angiogenic activity, and depending on the tumour and the drug or drugs administered, direct cytotoxic action on tumour cells or stimulation of the immune response, or both.

### 2.4 Is Metronomic Chemotherapy a New Therapeutic Option in Clinical Oncology?

The novel therapeutic approach of mct is emerging in the era of targeted cancer treatment. To date, several articles have been published reporting the use of mct in phase i or ii trials with drugs such as cyclophosphamide, doxorubicin, capecitabine, thalidomide, 5′-deoxy-5-fluorouridine, etoposide, paclitaxel, methotrexate, and prednisone, among others, with encouraging results.

The first work using mct in a clinical setting was published in 2002. Colleoni and colleagues evaluated the clinical efficacy of low-dose methotrexate and cyclophosphamide in heavily pretreated breast cancer patients, obtaining significant efficacy with minimal toxicity 65. Table I summarizes the clinical data reported since that publication. Some of the tumour types involved in the relevant studies were breast cancer 37,59,65–68,87, non-small-cell lung cancer 38, lymphoma 58,73, pediatric solid tumours 42,75,76, melanoma 78,79, and prostate carcinoma 82–84. The results so far obtained, some of which are very encouraging, clearly indicate that mct is worthy of further clinical evaluation.

In an interesting study, Bocci *et al.* performed a pharmacoeconomic evaluation of metronomic cyclophosphamide –methotrexate and a number of other novel phase ii regimens for the palliative treatment of metastatic breast cancer and concluded that the mct scheme could reduce health care costs, especially those associated with the combined use of the new highly expensive molecularly targeted therapies 87.

The preliminary evidence of disease stabilization obtained in patients with varying and progressing tumours, and the low toxicity profile registered when mct regimens are administered, are supporting mct implementation in the clinical setting with a predicted result of increased survival and life quality.

## 3. CONCLUSIONS

The data so far obtained induced us and other authors to begin a changing of our way of thinking about cancer treatment. To date, the aim of chemotherapy has been to achieve complete tumour suppression, a goal reached only exceptionally. Tumour elimination has been elusive, and metastasis accounts for an important part of this failure. We now know that all tumour cells cannot be consistently eliminated by high dosing schemes. The repeated administration of mtd, which induces important remissions, is generally followed by recurrences with the development of tumours even more malignant.

We can now focus on cancer therapy from a different angle. With low-dose chemotherapy, it may be possible to obtain a therapeutic effect in the clinical setting similar to that obtained experimentally. Should this hypothesis prove true, great progress will have made toward the solution of the cancer problem. On the other hand, present knowledge of tumour biology creates the recognition that tumour presence is not incompatible with patient survival. Therefore, it might not be necessary to aim for complete tumour eradication. A change from the apparently remote therapeutic objective of killing all tumour cells to the more pragmatic objective of diminishing tumour burden as much as possible and maintaining that diminishment over time can now be considered. This goal might be achieved by administering drugs in a low-dose metronomic schedule over time.

It is foreseeable that the knowledge acquired in the experimental field of mct, plus the increasing experience that is being obtained in the clinical setting, will spark a change in the way in which basic and clinical researchers design their therapeutic protocols against cancer.

## Figures and Tables

**TABLE I:** Clinical studies of metronomic chemotherapy

Reference	Treatment	Results
Breast cancer		
Colleoni *et al.*, 2002^65^	Cyclophosphamide plus methotrexate	2 cr, 10 pr, 8 sd; overall clinical benefit: 31.7%
Bottini *et al.*, 2006^66^	Letrozole plus cyclophosphamide	orr: 87.7%
Colleoni *et al.*, 2006^37^	Cyclophosphamide plus methotrexate, with or without thalidomide (thal)	Overall response: 20.9% without thal, 11.8% with thal
Orlando *et al.*, 2006^67^	Cyclophosphamide plus methotrexate	5 cr, 25 pr; 24 (15.7%) with prolonged clinical benefit
Orlando *et al.*, 2006^68^	Trastuzumab plus cyclophosphamide plus methotrexate	4 pr, 6 sd after 24 weeks; clinical benefit: 46%
Malignant vascular tumour		
Vogt *et al.*, 2003^69^	Pioglitazone plus rofecoxib plus trofosfamide	2 cr, 1 pr, 3 sd; median pfs: 7.7 months
Kopp *et al.*, 2006^70^	Trofosfamide	One case report: cr
Kaposi sarcoma		
Coras *et al.*, 2004^71^	Pioglitazone plus rofecoxib plus trofosfamide	One case report: pr and sd for 18 months
Non-small cell lung cancer		
Correale *et al.*, 2006^38^	Platinum plus etoposide	2 cr, 12 pr, 4 sd; overall response: 58.1%
Glioblastoma		
Kong *et al.*, 2006^72^	Temozolomide	pfs: 58.3% at 3 months
Lymphoma		
Buckstein *et al.*, 2006^58^	Celecoxib plus cyclophosphamide	2 cr, 9 pr, 6 sd; orr: 59%; median response duration: 8.2 months
Coleman *et al.*, 2008^73^	Prednisone plus cyclophosphamide plus etoposide plus procarbazine	10 cr, 8 pr; orr: 82%
Solid tumours		
Young *et al.*, 2006^74^	Cyclophosphamide, vinblastine plus rofecoxib	2 cr, 4 pr, 8 sd; orr: 30%
Pediatric solid tumours		
Stempak *et al.*, 2006^42^	Celecoxib plus vinblastine or cyclophosphamide	4 sd; orr: 13%
Sterba *et al.*, 2002^75^	Radiotherapy plus temozolomide	3 cr, 3 pr; orr: 75%
Sterba *et al.*, 2006^76^	Celecoxib plus 13-cisretinoic acid plus temozolomide plus etoposide	orr: 64%
Multiple myeloma		
Suvannasankha *et al.*, 2007^77^	Cyclophosphamide plus thalidomide plus prednisone	7 cr, 2 near-cr, 13 pr, 8 sd; orr: 86%
Melanoma		
Spieth *et al.*, 2003^78^	Treosulphan plus rofecoxib	1 pr and sd, 4 sd; orr: 60%
Reichle *et al.*, 2004^79^	Trofosfamide plus rofecoxib plus pioglitazone	1 cr, 1 pr, 2 sd; orr: 21%
Head and neck		
Caballero *et al.*, 2007^44^	Paclitaxel	1 cr, 20 pr; orr: 64%
Ovarian cancer		
Samaritani *et al.*, 2007^80^	Cyclophosphamide	One case report: pfs time: 65 months
Garcia *et al.*, 2008^81^	Bevacizumab plus cyclophosphamide	17 pr; orr: 24%
Prostate carcinoma		
Glode *et al.*, 2003^82^	Cyclophosphamide plus dexamethasone	psa level reductions: 9 patients ≥80%, 13 patients: 50%–79%, 2 patients: <50%; orr: 75%
Nicolini *et al.*, 2004^83^	Cyclophosphamide	2 pr, 3 sd; orr: 62.5%
Lord *et al.*, 2007^84^	Cyclophosphamide	orr: 34.5%
Colorectal		
Ogata *et al.*, 2007^85^	CPT-11 plus doxifluridine	Objective response rate: 36%; median overall survival: 452 days
Renal		
Krzyzanowska *et al.*, 2007 86	Cyclophosphamide plus celecoxib	1 pr, 6 sd; orr: 22%

cr= complete remission; pr= partial remission; sd= stable disease; orr= overall response rate; pfs= progression-free survival.
